# Finding Solutions for Fibrosis: Understanding the Innate Mechanisms Used by Super‐Regenerator Vertebrates to Combat Scarring

**DOI:** 10.1002/advs.202100407

**Published:** 2021-05-24

**Authors:** Fallon Durant, Jessica L. Whited

**Affiliations:** ^1^ Department of Stem Cell and Regenerative Biology Harvard University Cambridge MA 02138 USA; ^2^ The Harvard Stem Cell Institute Cambridge MA 02138 USA

**Keywords:** fibrosis, regeneration, vertebrates, wound healing

## Abstract

Soft tissue fibrosis and cutaneous scarring represent massive clinical burdens to millions of patients per year and the therapeutic options available are currently quite limited. Despite what is known about the process of fibrosis in mammals, novel approaches for combating fibrosis and scarring are necessary. It is hypothesized that scarring has evolved as a solution to maximize healing speed to reduce fluid loss and infection. This hypothesis, however, is complicated by regenerative animals, which have arguably the most remarkable healing abilities and are capable of scar‐free healing. This review explores the differences observed between adult mammalian healing that typically results in fibrosis versus healing in regenerative animals that heal scarlessly. Each stage of wound healing is surveyed in depth from the perspective of many regenerative and fibrotic healers so as to identify the most important molecular and physiological variances along the way to disparate injury repair outcomes. Understanding how these powerful model systems accomplish the feat of scar‐free healing may provide critical therapeutic approaches to the treatment or prevention of fibrosis.

## Graphical abstract

Fibrosis is a serious medical problem that remains unresolved in many cases. However, regenerative organisms have evolved to avoid fibrosis and are capable of scar‐free healing. Herein, the physiological and molecular mechanisms behind fibrosis and regenerative wound healing are reviewed and how this knowledge may be applied to solve issues in human health is explored.

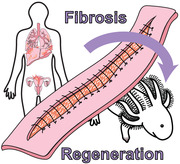

## 1 Introduction

Fibrosis is a serious medical problem that remains unresolved in many cases. Defined as excessive and aberrant deposition of extracellular matrix (ECM) tissue as a complication of disease or injury, this condition affects at least 100 million patients per year, only accounting for cases that follow surgeries in the developed world,^[^ ^1^ ^]^ and often results in debilitating or disabling health complications.^[^ ^2^, ^3^ ^]^ The ways that fibrosis can manifest in the body are vast; corneal scarring can lead to the loss of vision,^[^ ^4^ ^]^ pelvic adhesions are a leading cause of infertility, pregnancy complications, and bowel obstruction,^[^ ^5^ ^]^ and fibrosis of major organ systems can be fatal.^[^ ^6^ ^]^ Scarring of connective tissues such as ligaments, tendons, and the development of contractures over joints can be catastrophic to mobility and normal motor function.^[^ ^7^ ^]^ Fibrotic responses can also develop following orthopedic joint surgery or the placement of prosthetic implants, necessitating further surgical interventions in many cases. The psychological effects of these conditions can be severe,^[^ ^8^ ^]^ in some cases resulting in post‐traumatic stress disorder.^[^ ^9^ ^]^ Moreover, 45% of all deaths in the developed world are due to chronic fibrotic disease.^[^ ^10^ ^]^ Despite the many disparate means by which fibrosis can impact human health, the current understanding is that all of these pathologies may share a core cellular cause. For the purpose of this review, we will focus primarily on fibrosis occurring after injury, but many of these concepts will widely apply to other clinical incidents of fibrosis.

As an injury response, it has been suggested in the past that scarring is an evolutionary price we pay to heal wounds quickly.^[^ ^2^ ^]^ Yet, this idea is complicated by early human gestational fetuses and highly regenerative animals, both being capable of scar‐free regeneration after injury.^[^ ^11^, ^12^, ^13^, ^14^ ^]^ Current therapies for fibrosis in humans usually involve further injury through surgery to remove it, which is a gateway for repeat scarring upon healing. Scars can misdirect neural reconnections after injury^[^ ^15^ ^]^ and are considered to be a major impediment to regeneration.^[^ ^16^ ^]^ Animals that are able to regenerate have, through evolution, developed their own preventative and reparative strategies to combat scarring that have allowed them to perfectly restore the fidelity of their tissues postinjury. Therefore, novel approaches for future therapies may lie in understanding how these animals are able to antagonize fibrosis innately and how this differs from the postnatal human response to wounding. Some regenerative models that will be covered in this review include amphibians such as *Ambystoma mexicanum* and those from the *Xenopus* genus, the zebrafish *Danio rerio*, and mammalian models such as the *Mus musculus* digit tip and the spiny mouse of genus *Acomys*. It is also important to note that there is much to learn from human tissues that are able to heal scar‐free such as that of gestational fetuses and the oral mucosa. Through these models, not only may we uncover strategies to inhibit fibrotic response after injury, but we may also develop new insights for improving prospects for complex tissue regeneration in humans.

## 2 Overview of Scarring versus Regenerative Programs

**Figure 1:**
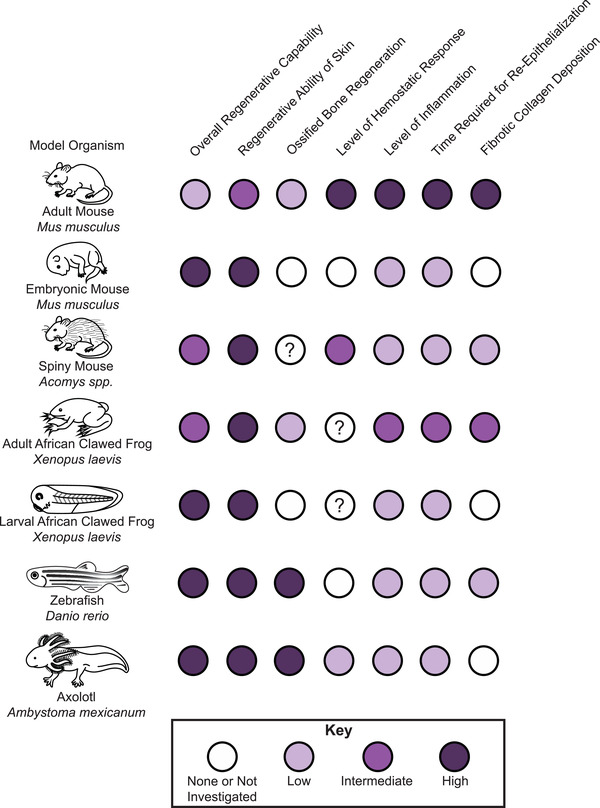
Regeneration and wound healing is variable across vertebrate organisms. Schematic showing representative regenerative abilities in different tissues, disparate time spent in the different stages of wound healing, and variability in cellular response across species. Progressively darker circles mean larger responses as indicated. White circles mean no response, whereas white circles with question marks indicate that the response has not yet been elucidated in the literature.

In order to understand how fibrosis due to injury occurs, it is important to understand the full wound healing response. The process of wound healing is generally divided into four phases. The first is the initiation of hemostasis whereby wounds are closed through clotting mechanisms. The second stage is an inflammatory stage. This stage allows the recruitment of important cell types that secrete factors that are crucial for the healing process as well as maintaining cleanliness in the area. Next, the area is rebuilt in the proliferation phase. Granulation tissue is formed, and re‐epithelialization occurs. The wound contracts and ECM proteins are laid down. The final phase is the maturation phase where the wound fully closes, collagen is remodeled, and collagen fibers are cross‐linked. The amount of time spent in each of these phases and the way they are executed are very different in organisms that experience fibrosis versus organisms that are able to heal without scars (**Figure** **1**). These details are addressed in detail in the sections that follow.

### 2.1 Hemostasis and Inflammatory Initiation

**Figure 2:**
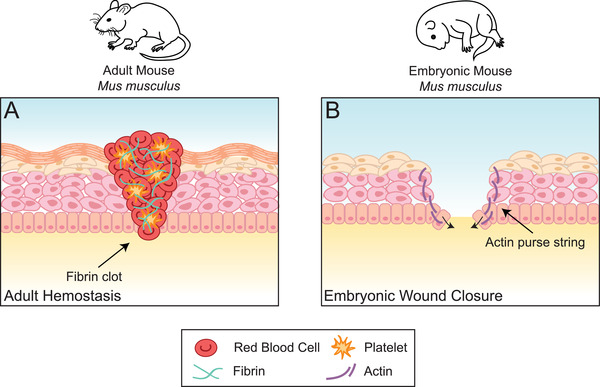
Wound closure is accomplished through different means in adult versus embryonic mice. A) The first stage of wound healing in adult mice is a hemostatic one, whereby the wound is closed with a fibrin clot. Damaged blood vessels lead to a coagulation of blood in the area of injury, activated platelets migrate to the site and form a sticky plug, and a fibrin network forms a mesh that ultimately creates a clot. B) In embryonic wounds, in lieu of a fibrin clot, at the leading edge of the wound epithelium, cells are connected to one another by actin filaments in a concentric circle around the wound. This cable acts as a contractile “purse string” that closes the wound without need of a coagulative cascade.

The first major step of an injury response is a hemostatic one, whereby a blood clot is formed to stop the flow of blood. In mammals, when blood vessels are injured, platelets are triggered to aggregate at the wound site. A clot made of cross‐linked fibers of fibrin then forms which stops the flow of blood, provides protection to tissues that are exposed by the wound, and serves as an early matrix containing embedded platelets that serves as a scaffold to support reparative cells.^[^ ^17^ ^]^ Although not much is known about how the coagulation cascade may impact the fibrotic response in injury‐induced fibrosis, it has been suggested that coagulation factors contribute to fibrosis of the liver, heart, and kidney.^[^ ^18^ ^]^ In the far more regenerative axolotl, in lieu of a true fibrin plug, a thin layer of coagulative cells forms at the wound bed and no scab forms^[^ ^12^ ^]^ (**Figure** **2**A). External fibrin clots are also not observed during zebrafish regeneration^[^ ^19^ ^]^ or embryonic wound healing^[^ ^20^ ^]^ whereby wound closure is accomplished by means of actin cables acting as a contractile “purse string”^[^ ^21^ ^]^ (Figure 2B). Even in late mammalian fetuses, although the hemostatic events are the same and occur in the same order as that of adults, these events occur more quickly and include larger amounts of fibronectin,^[^ ^22^ ^]^ which has been shown to accelerate and improve wound healing in other models.^[^ ^23^ ^]^

**Figure 3:**
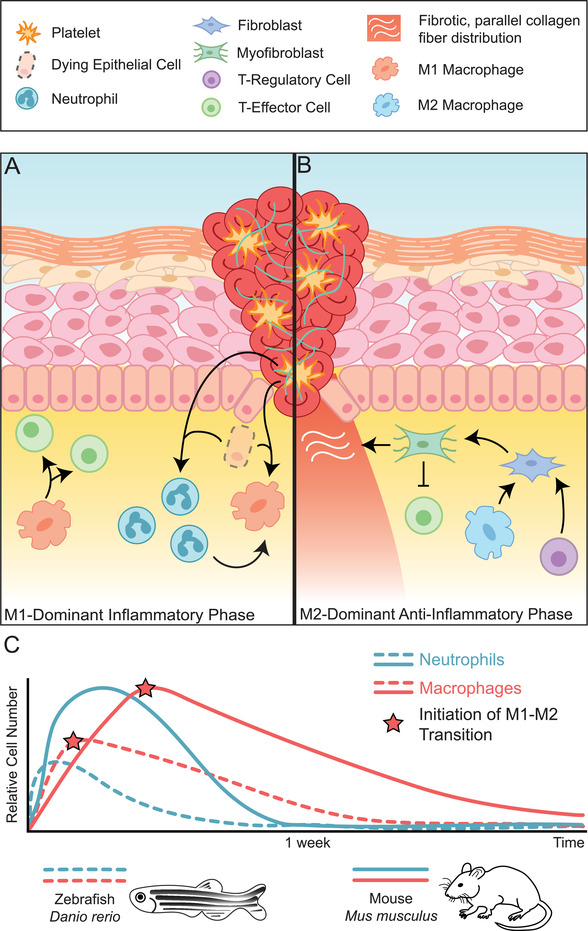
The phases of the inflammatory response to wound healing and differences observed in regenerative zebrafish versus non‐regenerative mouse. A) The proinflammatory response to wound healing is dominated by M1 macrophage polarization. In this phase, inflammatory M1 macrophages (orange), T‐effector cells (green), and neutrophils (teal) are recruited to the wound site. Signals released from dying epithelial cells and platelets drive this recruitment, encouraging neutrophils and monocytes to migrate into the area. M1 macrophages differentiate from monocytes, their polarization mediated by neutrophil signals. Macrophages then secrete cytokines that recruit T‐effector cells. B) An M2‐dominant phase begins when macrophages take on M2 polarization phenotypes (light blue) facilitated by T‐regulatory cells (purple). T‐regulatory cells and M2 macrophages secrete factors such as TGF‐*β* which leads to the differentiation of fibroblasts into myofibroblasts and the secretion of ECM and suppression of inflammatory T‐effector cells. C) The inflammatory response is markedly different in regenerative systems as observed in the zebrafish, *D. rerio* (dashed lines) than in the mouse *M. musculus* (solid lines). Comparatively, numbers of neutrophils (blue) and macrophages (red) are lower in zebrafish and peaks in relative cell number take place at earlier time points. The initiation of the transition from M1 to M2 polarization phenotypes in macrophages also take place earlier on (red stars).

The hemostatic differences during wound healing in more regenerative models are likely due to differences in wound‐induced inflammatory responses. These inflammatory responses are mediated by both the innate immune system, consisting of granulocytes, as well as lymphoid cells from the adaptive immune system, including T‐ and B‐cells.^[^ ^24^ ^]^ After adult mammalian injury, the inflammatory phase involves infiltration of a large variety of cells to the area including neutrophils, macrophages, and lymphocytes, which prevent infection. These cells are attracted to the area due to the release of inflammatory and remodeling factors by damaged tissue such as tumor necrosis factor‐*α* (TNF‐*α*), transforming growth factor‐*β*1 (TGF‐*β*1), matrix metalloproteinase‐9, and tenascin‐C^[^ ^25^ ^]^ as well as by the secretion of fibroblast‐modulating growth factors and cytokines by platelets embedded in the fibrin clot.^[^ ^26^ ^]^ Throughout the inflammatory process of wound healing, macrophages undergo a phenotypic transition from an M1 polarization to an M2 polarization.^[^ ^27^ ^]^ The transition from a monocyte‐derived M1‐dominant inflammatory phase (**Figure** **3**A) to the anti‐inflammatory M2‐dominant phase (Figure 3B) drives the changes in cell behavior that lead to different stages of wound healing. For example, in zebrafish caudal fin regeneration, M1‐macrophage‐mediated TNF‐*α* controls cell proliferation at the blastema, a mass of rapidly dividing cells that give rise to regenerated tissues, whereas M2 macrophages are involved in caudal fin remodeling.^[^ ^28^ ^]^ The M1–M2 transition in macrophage polarization takes place later in the timeline in mouse skin wound healing between 3 and 5 days post‐injury^[^ ^29^ ^]^ as compared to zebrafish caudal fin regeneration, where an increased presence of TNF‐*α* negative macrophages indicate this transition takes place around 20 hours post‐amputation^[^ ^28^ ^]^ (Figure 3C). The reverse transition from M2–M1 polarization dominance has been associated with loss of regenerative ability.^[^ ^30^ ^]^ Specifically, upon evaluation of the immune responses resulting from myocardial infarction in fetal mice, it was revealed that M2 macrophages are prominent during early, postnatal, regenerative periods, whereas M1 macrophages are prominent during later, postnatal non‐regenerative periods.^[^ ^31^ ^]^ This complements the observation that numbers of embryonic M2 macrophages decrease with age and are replaced by M1 macrophages in adults.^[^ ^32^ ^]^ It has been suggested that manipulating the polarization switch in macrophages directly at the wound site may accelerate healing,^[^ ^33^ ^]^ providing a potential approach for future therapeutics, especially for chronic wounds which are marked by an extended inflammatory phase. However, in many cases, the M1–M2 paradigm is not entirely straightforward given that M2 polarization has been linked to fibrotic disease such as pulmonary fibrosis.^[^ ^34^ ^]^

It is also important to note that recent literature has suggested that macrophages do not only play a role in the molecular cascade that eventually leads scarring: they also may contribute to scarring directly. In the zebrafish and mouse heart, after myocardial infarction, it was found that macrophages have the ability to directly contribute collagen to a forming scar that is separate from the collagen that is deposited by myofibroblasts.^[^ ^35^ ^]^ Therefore, the full scope of macrophage roles during wound healing is only now just being elucidated and there very well may be other direct macrophage functions during scarring or regeneration that are yet to be discovered.

Also of particular interest in this group of recruited inflammatory cells are neutrophils. Neutrophils are the first cells that are recruited to the wound site to clear bacteria and debris,^[^ ^36^ ^]^ but the relative number of neutrophils that arrive may be correlated with how proficient the animal is in scarless healing^[^ ^37^ ^]^ (Figure 3C). Oddly, classical studies showed that depletion of neutrophils does not dramatically alter wound healing in mammals,^[^ ^38^ ^]^ so it is still unclear how these differences in neutrophil infiltration may play a role in shifting scarring propensity. Rather than the presence or absence of neutrophils, perhaps it is an upstream process that impacts neutrophil production that is ultimately responsible for fibrotic or regenerative responses. Alternately, it could be behaviors of compensatory immune cells that result from a diminished presence of neutrophils, or changes in the actions of other immune cells that are typically stimulated by neutrophils that are responsible candidates for the downstream likelihood to generate fibrosis or regenerate.

Chemokines released by neutrophils attract macrophages to the wound site. This kickstarts phagocytosis to clear the area, followed by initiation of granulation tissue formation through the release of growth factors.^[^ ^26^ ^]^ What happens to these macrophages once the inflammatory phase of wound healing is complete is not entirely known, although it has been suggested that they may migrate away from the wound site^[^ ^39^ ^]^ or undergo apoptosis.^[^ ^40^ ^]^ Recently, it has been proposed that two‐thirds of fibroblasts present in granulation tissue come from myeloid cell lineage, most likely from these wound macrophages, induced by keratinocyte‐derived secretion of miR‐21.^[^ ^41^ ^]^ This suggests that the inflammatory phase is not only essential for providing the appropriate factors to support downstream wound healing processes, but it also provides major cellular support for ECM homeostasis. Given that fibroblast lineage is a factor in the determination of whether the composition of their secreted ECM will ultimately be fibrotic,^[^ ^42^, ^43^ ^]^ both the level of macrophage recruitment and rate of polarization could be a major driver for the differences in scar formation response in animals with varying immune responses.

T‐cells have also been implicated in regenerative processes. Regulatory T‐cells (T_reg_) in particular have been explored in regenerative systems due to their known anti‐inflammatory properties.^[^ ^24^ ^]^ In zebrafish, T_reg_‐like cells are required for regeneration of the spinal cord, heart, and retina.^[^ ^44^ ^]^ In amphibian models, suppressing the production of T‐cells delays limb regeneration in newts^[^ ^45^ ^]^ and it has been speculated that the refractory period, the developmental period whereby *Xenopus* tadpoles can no longer regenerate their tails, is at least partially due to the onset of T‐cell development.^[^ ^46^ ^]^ It is thought that the transition from M1–M2 macrophage polarization is also, at least in part, facilitated by T_reg_s,^[^ ^47^ ^]^ guiding wound healing systems away from proinflammatory environments toward anti‐inflammatory, restorative environments. Effector T‐cells recruited to the wound site have been implicated in fibrosis models. Specifically, Interferon‐gamma‐producing T‐cells have been shown to be involved in the activation of M1 macrophages, which contributes to the inflammatory response and cardiac fibrosis.^[^ ^48^, ^49^ ^]^ Most of the immune responses that are triggered by these cells are due to their release of various cytokines, but the effect that these cytokines have on fibrosis is tissue‐dependent.^[^ ^49^ ^]^

There are several major inflammatory signaling pathways that specifically contribute to fibrosis (reviewed in ref. ^[^ ^50^ ^]^). Of particular potency are TGF‐*β* and type 2 cytokines interleukin‐4 (IL‐4) and interleukin‐13 (IL‐13). TGF‐*β* is perhaps the most widely recognized driver of fibrosis due to its ability to induce *α*‐smooth muscle actin (*α*‐SMA) expression in fibroblasts, which pushes their conversion into myofibroblasts,^[^ ^51^ ^]^ the cells largely responsible for aberrant collagen production. This conversion has recently been shown to be dependent upon *calpain 9* and silencing this gene mitigates organ fibrosis in mice.^[^ ^52^ ^]^ Conversely, there are still no real explanations as to how this conversion coexists with TGF‐*β* also being important for scar‐free regeneration in many model systems,^[^ ^53^ ^]^ including axolotl,^[^ ^54^ ^]^ where there is no evidence it induces *α*‐SMA expression.^[^ ^11^, ^12^ ^]^ There is some evidence that relative levels of different TGF‐*β* isoforms make a difference in the regenerative response. In regenerative spiny mice *Acomys* spp., TGF‐*β*1 is relatively upregulated during wound healing compared with the fibrotic healer mouse, *M. musculus*.^[^ ^55^ ^]^ In fetal mouse and human wounds, profibrotic TGF‐*β*1 and TGF‐*β*2 are not highly expressed and TGF‐*β*1 is the most prominent in adult wounds.^[^ ^56^, ^57^ ^]^ Antifibrotic TGF‐*β*3, on the other hand, is highly expressed in rodent and human fetal wounds but not adult wounds, and ectopic TGF‐*β*3 aids in the treatment of scar tissue in adult rats.^[^ ^58^ ^]^

In terms of the type 2 cytokines, IL‐4 has been shown to contribute to ECM synthesis,^[^ ^59^ ^]^ and messenger RNA levels of IL‐4 are significantly higher in the fibrotic healer *Mus* than the regenerative *Acomys*.^[^ ^55^ ^]^ IL‐13 encourages fibroblasts to proliferate and differentiate in mice and induces gene expression of fibrosis‐promoting proteins.^[^ ^60^ ^]^ Other relevant cytokines that have been responsible for driving changes in fibrotic response include the proinflammatory interleukin‐6 (IL‐6) and interleukin‐8 (IL‐8) as well as the anti‐inflammatory cytokine interleukin‐10 (IL‐10). IL‐10 decreases IL‐6 and IL‐8 production, and IL‐6 and IL‐8 have been shown to be less prevalent in fetal human scarless healing,^[^ ^61^ ^]^ while in IL‐10‐deficient mice, fetal skin grafts are subject to scar formation.^[^ ^62^ ^]^ Moreover, overexpression of IL‐10 in adult mice decreases the inflammatory response and reduces abnormal collagen deposition.^[^ ^63^ ^]^ These immune factors are also expressed at different levels in neotenic versus metamorphic axolotls at different points throughout the regenerative period,^[^ ^64^ ^]^ suggesting these levels may play a role in the reduction of regenerative fidelity and rate in metamorphic animals. All in all, the presence of these factors and cytokines are starting points in understanding how the immune system modulates cellular behavior post‐injury and how these influences are linked to healing outcomes, but much work remains in using this information to impact human medicine.

Since the inflammatory response is crucial for the cellular response required for eventually laying down scar tissue, it may be tempting to suggest that reduced inflammation will drive healing responses away from fibrosis and toward regeneration. This hypothesis is especially appealing given that scar‐free healing in mammalian fetuses has been associated with a decreased inflammatory response^[^ ^61^, ^62^ ^]^ and differences in regenerative capability correspond with the respective immune system maturity.^[^ ^65^, ^66^ ^]^ Nevertheless, in animals capable of scarless healing, studies have agreed that inflammation is essential for regeneration. Macrophage‐depleted axolotls are able to heal limb amputation wounds, but they are unable to regenerate.^[^ ^67^ ^]^ Similarly, regeneration is not possible if macrophages are not available to support blastema proliferation in the zebrafish tail fin.^[^ ^68^ ^]^ This also has recently been applied to mammalian models showing that macrophages are necessary for regeneration in spiny mouse.^[^ ^69^ ^]^ Nonetheless, immune responses are not identical across all regenerative animals nor all scarring animals. It has been consistently shown across species that the length of time immune cells stay at the wound site is quite different in regenerative systems than in fibrotic systems^[^ ^26^ ^]^ (Figure 3C). Immune responses are complex and more nuanced aspects of how the immune system functions during wound healing in different species of varying regenerative abilities are only now just being explored. More research needs to be done on the ways that varying proinflammatory factors influence immune cell behavior as well as their resultant downstream consequences. Elucidating these processes would be a good first step toward the development of targeted immune therapies that drive wound healing toward effective tissue regeneration and away from both necrosis and fibrosis.

### 2.2 Proliferation and Re‐Epithelialization

**Figure 4:**
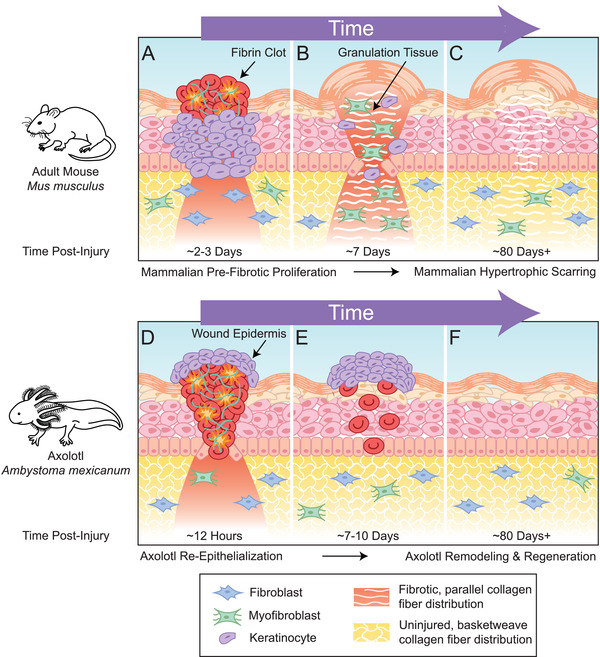
Differences between regenerative and non‐regenerative species in proliferation and remodeling stages of wound healing. A) Schematic representation of mammalian prefibrotic cellular proliferation in the adult mouse, *M. musculus* ≈2–3 days postinjury. Keratinocytes (purple) migrate to the area where the fibrin clot is located and begin to proliferate. These keratinocytes aid in breaking down the fibrin clot and make way for a provisional matrix. Fibroblasts (blue) also proliferate and migrate at this stage and the combined proliferative effort begins the formation of granulation tissue. B) Fibroblasts lay down ECM proteins and produce collagen and fibronectin replacing the fibrin clot with granulation tissue. Fibroblasts differentiate into myofibroblasts (green) which connect to the existing ECM and contract. C) The collagen that is deposited in the area takes on a new, parallel pattern that does not resemble the original basketweave pattern of uninjured tissue. The final result is hypertrophic scarring. D) Schematic representation of the amphibian re‐epithelialization and proliferation response in the axolotl, *A. mexicanum* ≈12 h postinjury. Keratinocytes crawl over the wound and proliferate after the wound has been covered to make a thick epidermis. Fibroblasts also enter the wound area, proliferate, secrete ECM, and some differentiate into myofibroblasts which similarly contract. E) The wound epidermis continues to thicken, and the clot begins to resolve, leaving behind some residual plasma, blood, and inflammatory cells. F) This ECM then undergoes extensive remodeling during regeneration that renders tissue indistinguishable from uninjured tissue including normal, basketweave collagen distribution. Adapted with permission^[^ ^26^ ^]^ Copyright 2018, Elsevier.

After inflammatory cells are recruited to the wound site, some of the most profound effects they impose on the wound healing process are through the release of fibroblast‐modulating growth factors and cytokines. This leads to a proliferation of fibroblasts which, coupled with the proliferation of keratinocytes, demarcates the proliferation phase of wound healing (**Figure** **4**A). The interactions between fibroblasts and keratinocytes have been shown to be crucial for wound healing.^[^ ^70^ ^]^ Keratinocytes and fibroblasts together synergistically promote the proliferative phase^[^ ^71^ ^]^ and guide the wound repair system out of the inflammatory phase and toward the establishment of granulation tissue and preliminary neovascularization. Excitingly, recent experiments have shown that stimulating both cell types together as a therapeutic option can reduce scarring in mouse burn wound models.^[^ ^72^ ^]^ In mammals, once fibroblasts are stimulated via molecular cues, largely TGF‐*β*,^[^ ^56^ ^]^ to differentiate to myofibroblasts, the wound contracts, and collagen production begins while keratinocytes are responsible for the process of re‐epithelialization. While most of this review discusses differences between animals that scar versus animals that regenerate, it is important to note here that wound closure does not even behave the same way in all scarring mammals. In “loose” skin mammals, such as mice and rats, alongside wound contraction mediated by myofibroblasts, wound closure is also initiated by panniculus carnosus muscle located in the subcutaneous tissue.^[^ ^73^ ^]^ It is thought that this muscle speeds wound healing and may reduce the need for extensive proliferation and re‐epithelialization that are experienced by “tight‐skinned” animals like humans and pigs.^[^ ^74^ ^]^

Each cell type has important functions on its own, though not all proliferative cells have the same behavioral trajectories. For example, when fibroblasts proliferate in adult mammalian wounds, they often transform to myofibroblasts that promote scarring. In regenerative animals, the proliferative cells in this stage of wound healing are blastema cells that are responsible for the replacement of lost tissue. The major question here that will guide the advancement of medicine is: what makes the difference that drives these proliferative cell types in the direction of regeneration versus fibrosis? Is it the molecular cues, the cellular interactions, the cellular lineage of the proliferating cells, or perhaps is it some combination of all the above?

#### 2.2.1 Role of Fibroblasts and Their Lineage in Wound Healing, Regeneration, and Scarring

After wounding in most adult mammals, fibroblasts migrate to the wound and begin to proliferate after receiving signals, mainly fibroblast growth factors (FGFs), TGF‐*β*, and platelet‐derived growth factor (PDGF) from the cells within the clot.^[^ ^75^ ^]^ Even this response could explain some differences between the way regenerative versus fibrotic systems operate as it has been observed that TGF‐*β* inhibits the proliferation of fetal human skin fibroblasts where it stimulates the proliferation in adults,^[^ ^76^ ^]^ and fetal wounds have been shown to express less PDGF than adult wounds.^[^ ^77^ ^]^ Zebrafish are highly regenerative, yet orthologous FGFs appear not to be required until after the wound closure stages.^[^ ^78^ ^]^

Mammalian wound sites become ripe with fibroblast activity whereby the fibroblasts begin to lay down ECM proteins and produce collagen and fibronectin. The resultant tissue replaces the clot that was a result of the coagulation cascade and is termed granulation tissue. Fibroblasts then undergo differentiation into myofibroblasts that express *α*‐SMA in adult mammals, which connect to the ECM and contract to reduce the surface area of the wound.^[^ ^79^ ^]^ Myofibroblasts then contribute to continued ECM deposition, promote the secretion of factors that allow for re‐epithelialization, neoangiogenesis, and the maintenance of the newly formed granulation tissue^[^ ^80^ ^]^ (Figure 4B). In the weeks that follow, the final result of injury manifests as collagen deposition and scarring (Figure 4C). Perturbing this process and elevating factors such as *mechanistic target of rapamycin complex 1* (*mTORC1*), which promote fibroblast proliferation and subsequent *α*‐SMA expression, has been shown to ultimately lead to increased collagen deposition and scarring.^[^ ^81^ ^]^ This has been confirmed in human disease whereby patients with mutations in the *p* *hosphatidylinositol‐4,5‐bisphosphate 3‐kinase catalytic subunit alpha* (*PIK3CA*) gene experience excessive scarring due to upregulations in the *p* *hosphoinositide 3‐kinase‐Protein kinase B‐mTOR* (*PI3K*–*Akt*–*mTOR*) pathway.^[^ ^82^ ^]^

Given these behaviors, it is not surprising that fibroblasts have primary roles in both regeneration and fibrosis. These functions are not species‐dependent; both are present in humans in different tissues and at different stages of aging. For example, the human oral mucosa is highly resistant to scar formation comparatively to that of human dorsal skin which is driven by fibroblast‐intrinsic properties.^[^ ^43^ ^]^ Similarly, during the gestational development of a fetus, the back skin transitions from being an organ capable of scarless regeneration to one that will fibrose.^[^ ^65^ ^]^ Using single‐cell fate mapping among other validations, it was determined that regenerative responses in mouse back skin are driven by *engrailed1*‐history‐naïve fibroblasts whose numbers reduce over time.^[^ ^83^ ^]^ These cells are then replaced by *engrailed1*‐history‐positive fibroblasts which drive scarring. This process can be rescued by transplanting *engrailed1*‐naïve cells, opening therapeutic opportunities that could potentially include grafting these cells to promote healing responses in lieu of fibrosis.

All of these data suggest that it is not the cellular environment that drives regeneration versus scarring, but rather the intrinsic nature of the cells that are present within that environment. However, there is new evidence that latent regenerative abilities can be induced in fibroblasts depending upon the microenvironment that they are exposed to.^[^ ^84^ ^]^ More specifically, reparative fibroblasts of quiescence‐associated factor *hypermethylated in cancer 1* (*Hic1*) lineage exposed to particular signaling environments that promote the reactivation of embryonic skin development genes leads to hair follicle neogenesis. Conversely, when the same cell types are exposed to alternative environments where these genes are not activated or suppressed, wound healing resembles a fibrotic healing pathway.^[^ ^84^ ^]^ This is hopeful for the progression of human medicine to combat fibrosis because it provides us another strategy from which we may tackle the problem of fibrosis. In addition to altering the genetic signatures of target cells or grafting restorative cells into affected areas, we may be able to change the behavior of fibroblasts regardless of their origin, by altering the wound environment with perhaps something as simple as a therapeutic drug cocktail.

There are very few instances in mammals where complete morphological fidelity can be restored following amputation without incidence of scarring. The most robust example of this phenomena that is currently studied is that of the digit tip, which is directly applicable to humans given that humans are able to regenerate the fingertip.^[^ ^85^ ^]^ Mice have classically been observed to regenerate the digit tip.^[^ ^86^ ^]^ In mice, after a wound‐healing response and inflammatory reaction generates a wound epithelium, a blastema forms which gives rise to a regenerated digit tip within 28 days post‐amputation.^[^ ^87^ ^]^ Recent experiments have demonstrated that this blastema is heterogeneous and broadly lineage restricted in terms of cell type.^[^ ^88^, ^89^ ^]^ Fibroblasts marked by *Paired related homeobox 1* (*Prrx1)*, an enhancer that has recently been implicated to demarcate a small subset of uniquely injury‐responsive adult dermal cells,^[^ ^90^ ^]^ are fate restricted to the mesoderm,^[^ ^89^ ^]^ and within the mesoderm, tissue‐specific progenitors regenerate the tendon, bone, and vascular endothelium. Single‐cell RNA‐seq analyses of the regenerating mouse digit tip have since revealed that fibroblasts constitute the most prominent and heterogenous population of cells in the digit tip blastema.^[^ ^91^, ^92^ ^]^ Several fibroblast markers were identified to be regeneration‐specific through these experiments including genes associated with inflammation (*C–C motif chemokine ligand 2* (*Ccl2*), *C‐X‐C motif ligand 2* (*Cxcl2*)), ECM regulation (*Matrillin 4* (*Matn4*), *Matrix* *metalloproteinase 13* *(* *Mmp13*)), and others with unknown molecular function (*Mesoderm* *‐specific transcript homolog protein* (*Mest*)) that will be excellent targets for future study.^[^ ^91^ ^]^ Other single‐cell RNA‐seq analyses in dorsal mouse wounds have also shown that injury induces heterogenous fibroblast populations that either differentiate toward myofibroblasts or nonmyofibroblast lineages.^[^ ^93^ ^]^ Moreover, some of these subsets express fibrosis‐related genes such as *platelet‐derived growth factor receptor alpha* (*PDGFRA*) or those associated with TGF‐*β* signaling. Distinct populations of fibroblasts have also been identified in human skin using single‐cell RNA‐seq techniques.^[^ ^94^ ^]^ Additionally, similar techniques have also revealed that fibroblasts that express the canonical Wnt transcription factor *Lymphoid enhancer‐binding factor‐1* (*Lef1*) have the ability to transform adult skin to a regeneration‐capable tissue.^[^ ^95^ ^]^ Further exploration of cell fate trajectories in regenerative mammalian contexts may suggest routes to inhibiting fibrotic pathways in favor of regenerative ones.

In nonmammalian, regenerative models, lineage relationships of heterogeneous blastema cells have been addressed to some degree,^[^ ^96^, ^97^, ^98^ ^]^ and it has been suggested by these studies that they may also be fate restricted. In the tadpole tail, spinal cord and notochord regenerate from the same tissue.^[^ ^98^ ^]^ In axolotl, using cellular labeling strategies, it was shown that regenerating muscle does not arise from cartilage cells and regenerating cartilage does not arise from muscle cells.^[^ ^96^ ^]^ In the zebrafish fin, osteoblasts and dermal fibroblasts were determined to come from separate lineages, arteries and veins come from the same lineage, and neuroectodermal cells also come from their own origin.^[^ ^97^ ^]^ In axolotls, single‐cell RNA‐seq analyses have also allowed for a better understanding of the cell types that compose blastemas.^[^ ^99^ ^]^ In zebrafish, outside of being the predominant cell type in regenerating fin tissue,^[^ ^100^ ^]^ fibroblasts also play an interesting role in the conversion of fibrotic response to a regenerative one in the injured heart. After cryoinjury that affects a quarter of the zebrafish heart, a fibrotic response consisting of ECM accumulation occurs followed by full regeneration.^[^ ^101^ ^]^ Through genetic ablation of the fibroblasts responsible for this ECM accumulation, it was discovered that these cells are actually necessary for the regenerative response and that they cease to produce fibrotic matrix as the heart heals under normal circumstances of injury.^[^ ^102^ ^]^ All of these data taken together suggest that although fibroblasts are heavily responsible for scar formation, they are also crucial for regeneration. Therefore, even though it may be tempting to develop therapies that attack the fibroblasts responsible for laying down unwanted ECM, it may be more beneficial to alter the behavior of these fibroblasts to better resemble a regenerative program instead. This strategy could not only be potentially beneficial for the prevention and treatment of fibrosis but could also potentially enhance the efficiency and integrity of wound healing.

#### 2.2.2 Role of Keratinocytes and Their Lineage in Wound Healing, Regeneration, and Scarring

Re‐epithelialization is initiated by a number of molecular factors such as epidermal growth factor (EGF) and TGF‐*α* that are secreted by keratinocytes as well as platelets and macrophages.^[^ ^103^ ^]^ This allows for epithelial cells to migrate to the wound site and begin epithelialization.^[^ ^104^ ^]^ Simultaneously, keratinocytes migrate to the area, begin to proliferate, differentiate, and express receptors that allow for them to interact with the ECM.^[^ ^105^ ^]^ Migrating keratinocytes are responsible for the phagocytosis that must occur to break down the fibrin clot and make way for a provisional matrix.^[^ ^103^ ^]^ The keratinocytes migrate over this matrix, underneath the fibrin clot, and hyperproliferate at the leading edge to re‐epithelialize the wound. The new epidermis becomes multilayered, and differentiation occurs to make a fully functional epidermis^[^ ^106^ ^]^ (Figure 4A). In regenerative species, the keratinocytes behave a bit differently in that they crawl over the clot (Figure 4D) and proliferate after the wound has been covered to make a thickened epidermis^[^ ^12^, ^26^, ^107^ ^]^ (Figure 4E). Re‐epithelialization consistently occurs at a much faster pace in regenerative systems^[^ ^26^ ^]^ (Figure 1). Even regenerative mammals like *Acomys* are able to close wounds much faster than non‐regenerative species.^[^ ^108^ ^]^

Dermal fibroblasts and epidermal keratinocytes are able to interact with one another at the dermal–epidermal junction (DEJ) to form a basement membrane of ECM.^[^ ^109^ ^]^ Macrophages have been demonstrated in several systems to stimulate production of connective tissue from fibroblasts and myofibroblasts.^[^ ^110^ ^]^ Less is known about the specific roles that keratinocytes play at the DEJ. Contrary to expectations, *Forkhead box‐O 1* (*FOXO1*), a transcription factor known to decrease proliferation and increase apoptosis,^[^ ^111^ ^]^ has been shown to promote wound healing by increasing keratinocyte migration and upregulating TGF‐*β*,^[^ ^112^ ^]^ indicating an important role in re‐epithelialization. Keratinocytes also contribute to the proliferation of fibroblasts and mesenchymal stem cells, the subsequent conversion to myofibroblasts, and downstream production of collagen as mediated by *FOXO1*.^[^ ^113^ ^]^ While loss of *FOXO* in *Drosophila* can rescue the poor regenerative ability of aging germline stem cells,^[^ ^114^ ^]^ whether modifying *FOXO*, keratinocyte interactions, or their downstream targets can enhance regeneration or scar‐free healing remains unclear.

Similarly, secreted factors from keratinocytes have been implicated in the development of skin fibrosis. A few of these secreted factors have been identified through the creation of a mouse model that overexpresses *Snail*, a zinc finger transcriptional repressor frequently upregulated in fibrotic tissues.^[^ ^115^ ^]^ When overexpressed in the basal keratinocyte layer of the skin, mouse skin manifests phenotypic representations of the fibrotic disease scleroderma.^[^ ^116^, ^117^ ^]^ This model leads to increased expression of secreted ECM protein *Fibulin‐5*, which stiffens tissues and aids in the activation of fibroblasts, creating a fibrotic feedback loop.^[^ ^117^ ^]^ Similarly, *plasminogen activator inhibitor type 1* (*PAI1*), which is highly expressed in fibrotic tissues, is also secreted by cells overexpressing *Snail*. *PAI1* plays an important role in fibrotic phenotypes due to its regulation of intracellular signaling in fibroblasts and its ability to increase mast cell infiltration. Mast cells are hematopoietic immune cells whose accumulation has been associated with fibrotic disease.^[^ ^118^ ^]^ *PAI1* activation leads to a cascade allowing for the binding of mast cells to fibroblasts, which, in turn, spurs fibrogenesis through increases in *α*‐SMA expression and mast cell degranulation.^[^ ^119^ ^]^ These examples underscore the large downstream cascades that keratinocytes contribute to in wide scale fibrotic processes.

### 2.3 Remodeling and Extracellular Matrix Deposition

#### 2.3.1 Transformation of the Wound Bed

After the proliferative phase of wound healing in mammals, the wound bed matures and is remodeled. At this point, myofibroblasts continue to secrete ECM, bind to collagen fibers, and contract. In the final stages of maturation, most of the fibroblast cells that were recruited to the wound site and proliferated there undergo apoptosis.^[^ ^120^ ^]^ Therefore, the amount of differentiation into myofibroblasts becomes extremely important: too little results in improperly healed wounds, too much results in excessive fibrosis.^[^ ^121^ ^]^ The initial collagen matrix that is laid to replace the provisional fibrin matrix is laden with collagen III that is subsequently degraded and replaced via collagen I synthesis.^[^ ^122^ ^]^ Collagen fibers at this stage are thicker and aligned in parallel, different from the basketweave pattern of uninjured tissue (Figure 4C); the parallel configuration in scar tissue causes reduced tensile strength and flexibility compared to normal tissue.^[^ ^123^ ^]^ Regenerative systems overcome this problem by producing elastin during regeneration,^[^ ^26^ ^]^ a protein that is not highly abundant in mammalian scar tissue.^[^ ^124^ ^]^ The collagen III:collagen I ratio is also higher in regenerative contexts, such as in fetal wounds^[^ ^125^ ^]^ and in the regenerative spiny mouse as compared with non‐regenerative *M. musculus*.^[^ ^126^ ^]^ Regenerated collagen tends to resemble uninjured tissue in that it re‐establishes a basketweave pattern (Figure 4F). Another striking difference between regenerative and non‐regenerative organisms at this stage is the timing of collagen deposition. In mouse, granulation tissue that forms by 4 days post‐injury is comprised of collagen and other ECM proteins,^[^ ^127^ ^]^ whereas appreciable collagen deposition is not observed until about 14 days post‐injury in axolotl.^[^ ^12^ ^]^ The transcription factor *Sal‐like protein 4* (*SALL4*), a gene that is important for maintaining stem‐cell‐like states during mammalian embryonic development,^[^ ^128^ ^]^ has been implicated in the control of timing of collagen deposition in axolotl.^[^ ^129^ ^]^ Two days post‐injury, *Sall4* is upregulated during wound healing in axolotl skin. Moreover, inhibition of *SALL4* leads to excessive collagen production during wound healing.^[^ ^129^ ^]^ Mammals do not upregulate *SALL4* after injury,^[^ ^130^ ^]^ which may contribute to some of these differences between species in the timing of collagen deposition. Future studies will have to resolve whether manipulating the timing of collagen deposition has the ability to alter fibrotic responses.

This stage of wound healing in regenerative models exhibits quite a different final profile of collagen remodeling. For example, in non‐regenerative species of rodents, collagen distribution consists of mainly thick fibers indicative of scarring, whereas regenerative mouse species like *Acomys* exhibit alternating thick and thin fibers in healed tissue that has similar architecture to that of uninjured tissue.^[^ ^131^ ^]^ Healed tissue in non‐regenerative rodents was primarily comprised of collagen I, and in regenerative rodents, the ECM is largely composed of fibronectin and tenascin. In regenerative amphibians like axolotl, deposited collagen is subject to extensive remodeling so that the final tissues resemble uninjured tissues (Figure 4F), unlike in most adult mammals where collagen retains a linear pattern in scar tissue (Figure 4C).^[^ ^129^ ^]^ This remodeling event that prevents long‐term scarring also occurs in zebrafish.^[^ ^132^ ^]^ Even when fibrosis is induced in axolotl through bleomycin injection, the fibrous‐like tissue that forms does not contain significant amounts of collagen and seems to be composed of mostly fibronectin.^[^ ^11^ ^]^ This suggests that a possible therapeutic option for pre‐existing scars may be to find a way to trigger a remodeling response in the collagen matrix of scars and promote a transition away from a primarily collagen‐I‐heavy distribution. Tunable collagen matrices consisting of nanocomposite hydrogels have been bioengineered to study physical properties of collagen networks,^[^ ^133^ ^]^ but manipulating collagen networks *in vivo* remains largely hypothetical. A good first step in the direction of this scientific advancement would be to develop a better understanding of why and how this collagen remodeling occurs in regenerative animals so that these molecular or cellular mechanisms can be applied to non‐regenerative systems like humans.

#### 2.3.2 Extrinsic Environment and Biomechanical Impacts on Scarring Response

The activation of myofibroblasts through conversion from fibroblasts is one of the most important components of a wound healing response. Their secretion and organization of the ECM provide the myofibroblasts the environment necessary to contract and close wounds. Nonetheless, when myofibroblasts are overactivated, this leads to the development of fibrosis through the overproduction of ECM components.^[^ ^134^ ^]^ The significance of tensile forces in driving the fibroblast‐to‐myofibroblast transition was recently explored through the use of 3D microtissues grown *in vitro*.^[^ ^135^ ^]^ In engineered clefts, fibroblasts transitioned to myofibroblasts at the growth front where the microtissue worked to close the cleft, modeling a closing wound site. This highly proliferative, contractile, tensed environment leaves an ECM underneath that matures into a homeostatic, relaxed, fibroblast‐rich environment created by the reversion of myofibroblasts to fibroblasts. Perhaps, one of the most interesting outcomes of these experiments was that this quiescent environment was not affected by the addition of supplemental TGF‐*β*1, which is known to promote the transition of fibroblasts to myofibroblasts. This further confirms that, in some contexts, tensile forces may serve as primary drivers in the maintenance of a myofibroblast‐heavy cellular environments that promote wound closure or fibrotic responses.

In the discussion of the proliferative phase of wound healing above, it was considered how dermal fibroblasts migrate to injured areas to lay new ECM onto the granulation tissue that is formed as a product of the coagulation cascade. However, more recently, new evidence has uncovered that skin scars may actually originate from pre‐existing matrix in the subcutaneous fascia that migrates along with fibroblasts to wound sites.^[^ ^136^ ^]^ Ablating the fascial fibroblasts that drag the facial matrix to the site of injury results in smaller scars with low collagen fiber and cell density. Similarly, placing a film underneath the wound to keep the migration from occurring leads to chronic open wounds. The properties of superficial and subcutaneous fascia vary among anatomical location and across species^[^ ^137^ ^]^ and could potentially explain variations in the propensity to scar. Complementarily, recent experiments have suggested that the microarchitecture of collagen may directly influence the differentiation of myofibroblasts by biomechanically modulating cell signaling.^[^ ^138^ ^]^ It was shown that adipose stromal cells cultured in scaffolds with thicker fibers had more contractile properties, expressed the markers of myofibroblasts, and also deposited more fibronectin fibers. Given that a wide variety of clinical studies have suggested that fascial stiffness can be altered due to the presence of contractile cell types like myofibroblasts,^[^ ^139^ ^]^ direct manipulation of the behavior of these cells in areas such as the fascia before controlled injuries like surgery may be an effective fibrosis preventing strategy. It has also been identified that myofibroblasts are plastic enough to be reprogrammed through exposure to growth factors like bone morphogenic protein (BMP). Specifically, when keloid fibroblasts were treated with BMP, adipocytes formed in place of scarring,^[^ ^140^ ^]^ again suggesting that myofibroblast manipulation may be an effective fibrosis treatment strategy.

Despite the evidence that biomechanics are instructive for wound healing and scarring, studies currently documenting matrix biology and tissue stiffness during healing in highly regenerative animals are not extensive, especially *in vivo*. In zebrafish, gene profiling studies have recently focused on basement membrane gene expression kinetics during caudal fin regeneration.^[^ ^141^ ^]^ It was found through these analyses that the profile of the basement membrane recapitulates what is found during development, specifically through the re‐expression of the embryonic *col14a1a* gene, which is important for proper timing of laminin deposition. Although it was found that knocking out this gene leads to an increase in the stiffness of the basement membrane, there are no apparent impacts on regenerative capability. Mammalian *in vitro* experiments using *Acomys* dermal cells have uncovered that stiffer substrates do not promote myofibroblast activation or the assembly of many *α*‐SMA‐positive stress fibers as they do in *Mus* dermal cells.^[^ ^142^ ^]^ Ultimately, in order to garner a robust understanding of how extrinsic biophysical factors can impact regeneration and guide the development of antagonistic therapies for fibrosis, more *in vivo* experiments in highly regenerative animals may be needed to elucidate the role of biomechanics during regeneration, blastema formation, and wound healing.

## 3 Impact of Aging and Metamorphosis on Fibrosis

Given the strong influence the extrinsic environment has on the generation of fibrosis at a cellular level, it is not surprising that greater macrolevel factors, such as aging and metamorphosis, within the same organism drastically alter fibrotic response. In this review, we have already discussed some of the classically observed differences between fetal and adult wound healing, but more recently, the impact of aging on fibrosis has been explored on a molecular and cellular level. Interestingly, the correlation between the likelihood to generate fibrosis and age is not linear and will require a deeper understanding of how fibrosis mechanistically changes throughout aging. This understanding would allow us to take advantage of age‐dependent factors that innately combat fibrosis and also develop treatments that are specific to patients of varying age populations.

The majority of general assumptions in the field have held that the integrity of tissues declines with age which makes the ability for these tissues to repair more challenging. With age, phenotypic changes are obvious, including skin wrinkling, thinning, and lack of rigidity. Biologically, this likely stems from decreased thickness of the dermis, ECM density, and fibroblast population numbers.^[^ ^143^ ^]^ Transcriptomics studies have revealed that dermal fibroblasts struggle to retain their heterogenous identities as they age. More specifically, they begin to produce less dermal ECM and more products that are reminiscent of what one would expect from that of adipocytes.^[^ ^144^ ^]^ This is problematic because the production of ECM is crucial for skin repair; however, it is also the foundation of fibrotic responses. It was found that some of the transcriptional changes in aging cells can be abated using dietary changes; but, it was variable how stable these modifications were, and old dermal fibroblasts were always distinguishable from young dermal fibroblasts.^[^ ^144^ ^]^ Ultimately, this research shows us that cell identity and how it relates to the fibrotic properties of the skin is plastic and it may be a matter of identifying how factors like metabolism and the environment can play a role in driving cell behavior in particular desired directions.

The concept that tissue repair universally declines as a result of age is not entirely true. It is also important to note that there is some benefit to the fact that the elderly heal with observably thinner scars than younger individuals and are less likely to form keloid and hypertrophic scars.^[^ ^145^ ^]^ Inevitably, thicker scars are more problematic than thinner scars not only because of their appearance, but in their increased likelihood to disrupt other important biological tissues like connective tissues, joints, and nerves. Wound repair takes place at a slower pace in older animals,^[^ ^146^ ^]^ but the integrity of the skin itself is not necessarily compromised.^[^ ^147^ ^]^ Researchers were able to determine through parabiosis that there must be a fibrosis‐promoting, circulating factor in young mouse blood because when exposed to young mouse blood, elderly mice no longer regenerated as robustly. *Stromal‐derived factor 1* (*SDF1*), a conserved appendage regeneration gene,^[^ ^148^ ^]^ is expressed highly in young mice and promoted skin regeneration in young mice upon deletion. In older animals, *enhancer of zeste homolog 2* (*EZH2*), which prevents *SDF1* activation, is more readily recruited to the *SDF1* promoter, a genomic observation also seen in human wound healing.^[^ ^130^, ^149^ ^]^ By blocking *EZH2* function pharmacologically, *SDF1* was reinduced in elderly mice and they no longer could regenerate as profoundly.^[^ ^150^ ^]^ This work will lead to a promising pursuit of a scar‐preventative clinical trial with Food and Drug Administration‐approved *SDF1* inhibitors.

Aside from gross physiological aging, it is also likely that cellular senescence plays a role in guiding a healing system toward fibrosis versus regeneration. One major difference that was identified between regenerative species of mice (*Acomys*) and those that produce fibrosis (*Mus*) was a lack of cell proliferation at the local level in species with a greater propensity to scar.^[^ ^131^ ^]^ Although this correlation was shown to not be consistent across all fibrosis producing rodent groups (*Rattus*), it was shown with consistency that regenerative species were more resistant to cellular senescence when exposed to oxidative stress.^[^ ^151^ ^]^ Injury‐induced reactive oxygen species (ROS) have been documented to be crucial for regeneration in multiple species,^[^ ^152^ ^]^ but this new evidence shows that the downstream impacts that ROS have on cellular senescence may be what drives fibroblast behavior either toward regenerative healing or increased collagen production and scarring.

**Figure 5:**
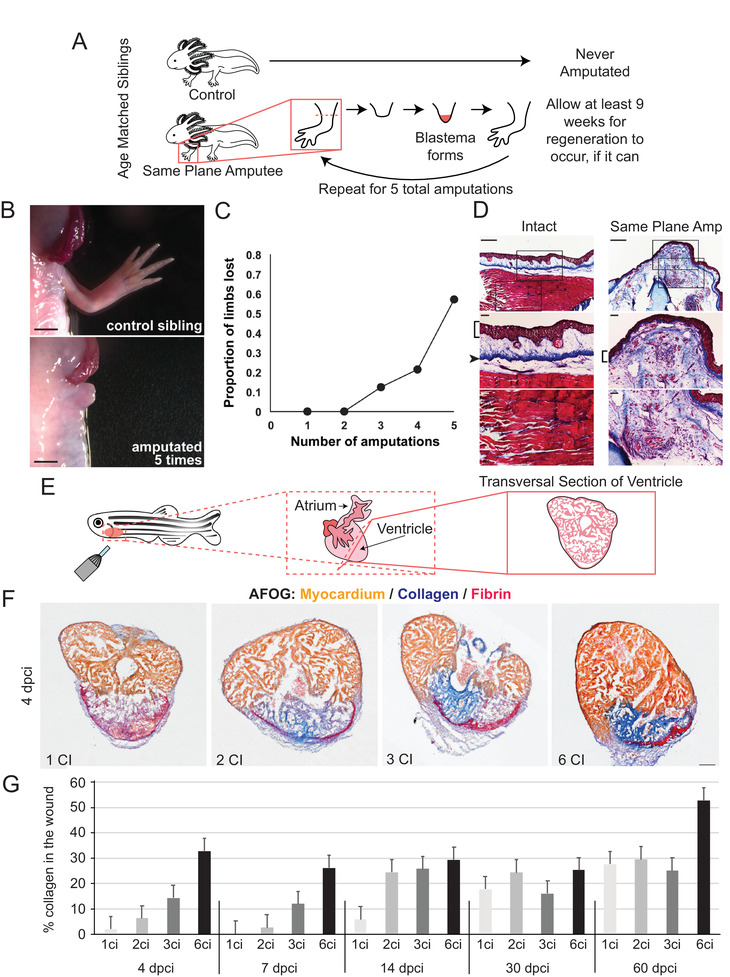
Regenerative decline and fibrosis after repeat injury in axolotl and zebrafish. A) Experimental overview – age matched siblings were used for repeat amputation experiments whereby control animals were never amputated and experimental animals underwent amputation of both forelimbs. The limbs were allowed at least 9 weeks to regenerate, if they were able, and were then challenged to a repeat amputation in the same amputation plane. This process was repeated until the animal was subject to 5 total rounds of amputation. B) Representative bright‐field photos of the control sibling limb (left) and a limb that failed to regenerate in an experimental animal after repeat amputation (right). C) Cumulative distribution plot of loss of the ability to regenerate beyond the plane of amputation. D) Limb stumps that fail to regenerate exhibit persistent collagen deposition, as observed with Masson's trichrome stain. Intact specimen with no amputations (left) shows normal collagen distribution. Failed regenerates following a repeat same plane amputation (right) showed extensive scar tissue, as evidenced by collagen deposition proximal to the plane of amputation. Middle and lower panels are higher magnification views of the images in the top panels. Brackets indicate epidermis, arrowhead indicates dermis in the control. Scale bars in the top panels are 500 µm and scale bars in the middle and bottom panels are 100 µm. B‐D) Adapted with permission.^[^ ^153^ ^]^ Copyright 2017, The Authors, published by Nature Portfolio. E) Representative schematic of cryoinjury in zebrafish, *D. rerio*. F) Representative transversal sections of regenerating, cryoinjured zebrafish hearts at 4 days post‐cryoinjury stained with acid Fuchsin and Orange‐G (AFOG) reagent showing myocardium (beige), fibrin (red), and collagen (blue). Multiple cryoinjuries show more collagen deposition. Scale bar = 100 µm. G) Histogram depicting the percentage of collagen observed in the wound area after cryoinjury. Early in regeneration, the amount of collagen deposition is progressive with increasing numbers of cryoinjury exposure. *N* ≥ 4 hearts, 3 sections per heart. F,G) Adapted with permission.^[^ ^154^ ^]^ Copyright 2020, The Authors, published by Springer Nature.

Although it is unlikely due to cellular senescence, it has also been observed that axolotls exposed to repeated amputation as they age over time seems to exhaust their capability to regenerate and instead exhibit fibrotic phenotypes^[^ ^153^ ^]^ (**Figure** **5**). After undergoing up to five rounds of amputation within the same plane of the limb (Figure 5A), animals experienced a severe reduction in their ability to initiate limb regeneration (Figure 5B,C). Histological observations revealed abnormal collagen deposition in these animals, indicating the presence of fibrosis (Figure 5D). Using RNA‐seq, a number of differentially expressed transcripts were identified in the animals who underwent repeat amputation as compared with their sibling‐matched controls. One of these genes was the EGF‐like ligand *amphiregulin*, and its misexpression leads to thickened wound epidermis, delayed regeneration, and regenerative defects in naïve axolotls upon primary amputation. These limitations in regenerative response after multiple injuries have also been recently observed in zebrafish cryoinjury models. Normally, zebrafish have the ability to restore damaged ventricles after cryoinjury and serve as a common injury model used to demonstrate zebrafish heart regeneration (Figure 5E); however, after six cryoinjuries to the heart, the animals are no longer able to resolve fibrotic tissue that manifests after injury.^[^ ^154^ ^]^ After one, two, and three cryoinjuries, similar levels of regenerative capability were observed; however, after two months of regeneration, animals exposed to six cryoinjuries were left with wounds that were nearly twice the size of the other groups.^[^ ^154^ ^]^ Moreover, wound sites up to a week old contained much more collagen after multiple cryoinjuries as compared with one cryoinjury (Figure 5F). In fact, the amount of collagen that was deposited after repeat cryoinjuries was progressive. A week after a single cryoinjury, collagen deposition was seen in only 1% of the area of injury. With each cryoinjury, this percentage would increase: 5% after two cryoinjuries, 15% after three cryoinjuries, and 30% after six cryoinjuries^[^ ^154^ ^]^ (Figure 5G). These data suggest that fibrotic responses are induced early on in the regenerative response in the case of repeat cryoinjury. Similar restrictions to regenerative ability post‐injury have been detected in the zebrafish retina and maxillary barbel.^[^ ^155^ ^]^ The full molecular mechanisms behind these kinds of regenerative impairment have yet to be elucidated, but these fibrotic phenomena occurring after repeat injury represent further examples of how a once‐regenerative system can be pushed into fibrotic pathways. Since powerful regenerative systems can be pushed toward fibrosis, understanding the molecular mechanisms behind this shift may suggest development of therapeutics or physiological manipulations that could inhibit fibrotic responses in humans.

There are potentially parallels worth exploring between the relationship of aging with fibrosis and the relationship of metamorphosis with decreased regeneration capability or speed. Although humans are not traditional metamorphic animals, the highly regenerative fetus must go through immense transformation before birth, when the predisposition to scar becomes much stronger. Impressively, even though regeneration may take place at a slower pace, postmetamorphic newts and axolotls are still able to heal scar‐free.^[^ ^12^, ^13^ ^]^ Therefore, the major question remains, what molecular or physiological mechanisms are these animals using that enable them to undergo this massive transformation and still retain their proclivity to combat scarring? Are these mechanisms lost in humans and other animals who fibrose or fail to regenerate once they are out of the fetal or premetamorphic period? These kinds of questions were thoroughly reviewed in a comparison of scar‐free repair in frogs versus salamanders.^[^ ^13^ ^]^ Salamanders maintain their scar‐free healing capabilities, while frogs (*Xenopus*) lose their ability to regenerate appendages after metamorphosis, resulting in the formation of cartilaginous spikes.^[^ ^156^ ^]^ This review suggests that the most prominent difference between these two systems may be immunological in nature whereby the premetamorphic immature immune system in anurans is responsible for supporting scar‐free healing responses, similar to what is observed in mammalian fetuses.^[^ ^65^, ^66^ ^]^ In *Xenopus*, perturbing other mechanisms such as membrane voltage^[^ ^157^ ^]^ and brief application of exogenous progesterone^[^ ^158^ ^]^ have been shown to improve regenerative outcome in non‐regenerative stages. Whether these manipulations change downstream immune responses *in vivo* to result in the improved regenerative outcomes, as it has been shown in both *Xenopus* ^[^ ^159^ ^]^ and other systems,^[^ ^160^ ^]^ or whether there is another unknown pathway that is ultimately responsible, remains to be determined.

## 4 Translating into Clinical Models

Understanding fibrotic behavior from a biological perspective is incredibly important and will ultimately lead to the development of new tools that treat and prevent scarring in human patients. Research on the molecular and cellular foundations of fibrosis has begun to spawn creative clinical treatment models. Perhaps, one of the most surprising therapeutics for skin wounds that has been introduced in recent years is the potential use of snail mucus as a healing agent.^[^ ^161^ ^]^ *Helix aspersa muller* mucus has been used as a dermatological serum since ancient times and more modernly has been used as a pharmaceutical for wound management and respiratory ailments.^[^ ^162^ ^]^ Recently, this compound has been more thoroughly characterized through *in vitro* experimental models and has shown to aid in the proliferation and migration of fibroblasts.^[^ ^161^ ^]^ Future *in vivo* experiments will reveal whether these substances or extractions thereof have any impact on scar reduction or improvements to healing chronic wounds.

Other clinical models that are currently in development have not had quite as serendipitous of a discovery story and have come as a result of rigorous expansions upon the molecular and cellular fibrosis literature. Many of these expansions require a good medium for preclinical drug screening and testing given that fibroblasts behave differently when they are plated on plastic surfaces than they do in an *in vivo* system.^[^ ^163^ ^]^ The most common solution to this problem is through the use of 3D culture;^[^ ^164^ ^]^ however, new strategies involving macromolecular crowding have provided a helpful simulation of crowded *in vivo* cellular environments which reduces some of the lengthy culture times imposed by other systems.^[^ ^165^ ^]^ This approach has created a model for skin fibrosis that has revealed that fibrotic responses vary depending upon what stimulates its formation.^[^ ^166^ ^]^ More specifically, the ECM profiles and phenotypes that represent the hallmarks of fibrosis in this system are very different whether they are induced by TGF‐*β*, PDGF, or IL‐6.^[^ ^166^ ^]^ It is hopeful for rapid drug screening purposes that this system was effective for identifying a number of drugs that are specific for each of these fibrotic stimulants. This suggests that not all fibrosis is alike and may need to be treated differently depending on the patients, their age, and composure of their scarring phenotype.

One of the most promising clinical therapies that is being exploited broadly to treat medical issues is that of T‐cell immunotherapy. The most common use of this promising technology is in the treatment of cancer,^[^ ^167^, ^168^ ^]^ but has also been proposed for the treatment of fibrosis.^[^ ^169^ ^]^ This type of immunotherapy is achieved by redirecting T‐cells by encouraging them to localize to specific antigens on the desired cell type that needs to be eliminated using either modified T‐cell receptors^[^ ^170^ ^]^ or chimeric antigen receptors.^[^ ^167^ ^]^ In models of cardiac fibrosis, targeting cardiac fibroblasts through genetic ablation has shown effectiveness in the past;^[^ ^171^ ^]^ therefore, these cardiac fibroblasts are considered to be good candidates for immunotherapy targets for the treatment of cardiac scarring. Combining these scientific pursuits, researchers were able to reduce cardiac fibrosis and its ill effects on the heart through adoptive transfer of CD8^+^ T‐cells that were specific for antigens specific to fibroblast activation protein in injured mice. There is still much work that needs to be done to minimize potential off‐target effects and determine what the optimal immunotherapy targets for varying manifestations of fibrosis would be. Only once these important factors are elucidated can this potentially powerful therapeutic strategy be applied to humans in a clinical space. Nonetheless, these experiments have shown an excellent proof of concept that immunotherapy may be an effective way to treat or prevent fibrotic disease.

## 5 Final Considerations and Future Perspectives

**Figure 6:**
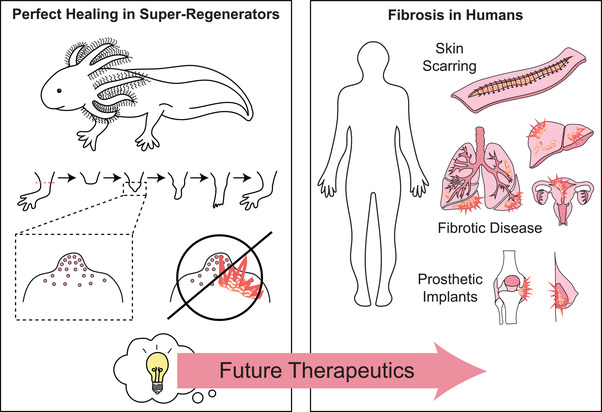
Utilizing the knowledge garnered from super‐regenerators to solve the human health problem of fibrosis. It is well documented that there are animals, such as the depicted axolotl, *A. mexicanum*, that have the capability to heal scarlessly. Understanding the molecular mechanisms behind how they are able to accomplish this natural feat will lead to important ideas that will guide future therapeutics for the treatment and prevention of human fibrotic disease. This is a serious medical problem that is need of good solutions due to its manifestations in outward scarring of the skin, fibrotic disease of organ systems such as the liver, lung, and uterus, and its contribution to prosthetic implant failure. Effective therapeutics accomplishment would alleviate the pain and suffering of many millions of patients per year and would even have the potential to save lives.

The study of regenerative models provides a unique opportunity to solve one of the most important medical problems that we have in human health: fibrosis. Many animals, and even some human tissues, have the capacity to heal fully scar‐free. This means that a genetic blueprint exists that will allow for regenerative healing in place of fibrosis and discovering how to activate these pathways that are innately present is likely to be key for future therapeutics (**Figure** **6**). Aside from cosmetic benefits, resolving fibrosis would alleviate severe psychological and physical problems within the patient population. It would prevent a tremendous number of surgeries from having to be repeated and, in the case of severe organ fibrosis, would even save lives. Regenerative healing also likely lies on a continuum between fibrosis and chronic wounds that struggle to repair, so by understanding these processes in greater detail, we may have a better chance at developing effective therapeutics for necrosis and ulcers as well. It remains to be seen whether the best strategy will be to develop therapies that focus on modifying molecular factors at the wound site, transplanting cells of particular lineages into the area, encouraging ECM realignment once wound healing is complete, or another approach inspired by research in this fast‐changing field. The future of fibrotic research is an exciting one and may even lead to the larger goal of enhanced regenerative response in humans after large tissue loss and amputation.

## Conflict of Interest

F.D. declares no conflicts of interest. J.L.W. is the co‐founder of Matice Biosciences.
